# Visual outcomes and safety of an extended depth-of-focus intraocular lens: results of a pivotal clinical trial

**DOI:** 10.1097/j.jcrs.0000000000000747

**Published:** 2021-07-13

**Authors:** Daniel H. Chang, Devi Priya Janakiraman, Pamela J. Smith, Anne Buteyn, Joy Domingo, Jason J. Jones, William C. Christie

**Affiliations:** From the Empire Eye and Laser Center, Bakersfield, California (Chang); Johnson & Johnson Vision, Santa Ana, California (Janakiraman, Smith, Buteyn, Domingo); Jones Eye Clinic, Sioux City, Iowa (Jones); Scott & Christie and Associates, PC, Cranberry Township, Pennsylvania (Christie).

## Abstract

To compare the effectiveness and safety of the TECNIS Symfony intraocular lens (IOL; ZXR00) with the TECNIS 1-piece monofocal IOL (ZCB00).

**Setting:**

15 sites in the United States.

**Design:**

Prospective, randomized, patient-masked/evaluator-masked clinical trial.

**Methods:**

Randomized participants received either the ZXR00 or ZCB00 IOL bilaterally. The 6-month postoperative outcomes included monocular and binocular distance, intermediate, and near visual acuity (VA), spherical equivalent refraction and refractive cylinder, spectacle wear, and visual symptoms.

**Results:**

Overall, 299 patients were implanted with a study IOL (ZXR00 IOL, n = 148; ZCB00 IOL control, n = 151). At the 6-month follow-up, mean binocular uncorrected distance VA was comparable between ZXR00 and ZCB00 IOL recipients (*P* = .1011). The ZXR00 IOL group had significantly better mean binocular uncorrected intermediate VA and uncorrected near VA (both *P* < .0001) than the ZCB00 IOL group. The mean binocular distance-corrected intermediate VA and distance-corrected near VA were also better in the ZXR00 IOL group (both *P* < .0001). More ZXR00 IOL recipients reported wearing spectacles none of the time or a little of the time for overall vision at 6 months compared with the ZCB00 IOL group (85.0% vs 59.9%, *P* < .0001). In the ZXR00 IOL–implanted patients, low incidence rates of night glare (mild to moderate, 2.7%), halo (mild to moderate, 13.6%; severe, 2.7%), and starbursts (mild to moderate, 7.5%; severe, 1.4%) were reported.

**Conclusions:**

The TECNIS Symfony IOL provided comparable distance vision and improved uncorrected and distance-corrected intermediate and near vision, along with decreased spectacle wear and low incidence rates of dysphotopsia, compared with the TECNIS 1-piece monofocal IOL.

## Abstract 2

Randomized comparison of the TECNIS Symfony (ZXR00) and the TECNIS 1-piece monofocal (ZCB00) intraocular lenses in cataract surgery demonstrates improved intermediate and near vision with TECNIS Symfony.

Current clinical options for patients with cataracts desiring improved vision across a range of distances include a choice of monovision or multifocal intraocular lenses (IOLs). Patients implanted with standard monofocal IOLs often need spectacles for reading or performing other near tasks, even if a monovision option is selected.^1,2^ Common spectacle options, including bifocal and progressive addition lenses, can increase the risk for trips and falls in the elderly population.^3^ Patients implanted with multifocal IOLs are able to read and perform other near tasks without spectacles, but they sometimes experience dysphotopsia (eg, halos and glare), particularly at night, and have limitations in intermediate vision.^4,5^ Accommodating IOLs are available, although their effect can depend on fit within the capsular bag or capsular bag elasticity, and results have been less predictable.^6,7^

The TECNIS Symfony extended depth-of-focus (EDoF) IOL (Johnson & Johnson Vision) incorporates a diffractive echelette design that elongates the focus, creating a continuous range of vision. Unlike multifocal IOLs that split the light into distinct focal points, the elongated focal zone of EDoF IOLs reduces overlap of near and far images, thus generating a lower incidence of halos and glare.^8,9^ The use of achromatic technology to correct longitudinal chromatic aberrations improves visual performance of the EDoF IOL.^8,9^

This pivotal clinical trial of the TECNIS Symfony IOL (model ZXR00) was designed to evaluate its effectiveness and safety compared with a monofocal IOL, the TECNIS 1-piece IOL (model ZCB00).

## METHODS

### Study Design

This prospective, bilateral, randomized, comparative, patient-masked/evaluator-masked, multicenter study was conducted at 15 sites throughout the United States (ClinicalTrials.gov; NCT02203721). The study was initiated in August 2014 and completed in June 2015. All patients provided written informed consent, and the approval was obtained from the U.S. Food and Drug Administration and institutional review board. The study was conducted in accordance with the U.S. Code of Federal Regulations, the tenets of the Declaration of Helsinki, and all other applicable laws and regulations.

### Inclusion and Exclusion Criteria

Patients were included in the study if they were 22 years or older with bilateral cataracts for which phacoemulsification extraction and posterior chamber IOL implantation were planned. Each eye had a preoperative corrected distance visual acuity (CDVA) of 20/40 Snellen or worse with or without a glare source, potential for postoperative CDVA of 20/30 Snellen or better, normal corneal topography, preoperative corneal astigmatism of 1.00 diopter (D) or less, and clear intraocular media other than cataract. Patients were required to sign an informed consent form and HIPAA of 1996 authorization and be able to understand and respond to a written questionnaire.

Key exclusion criteria precluded eligibility for the following reasons: any previous ocular trauma, ocular surgery, ocular or systemic condition, or degenerative disorder that could affect visual outcomes or increase risk, previous corneal refractive surgery, use of systemic or ocular medications that could affect vision, inability to focus or fixate for prolonged periods, any condition associated with the fluctuation of hormones that could lead to refractive changes, or participation in any other clinical trial during or 30 days before the preoperative visit.

### Study IOL Description

The 2 IOLs compared in this study were the investigational TECNIS Symfony IOL (model ZXR00) and the TECNIS 1-piece monofocal IOL (model ZCB00). The ZXR00 is a diffractive, aspheric, foldable, acrylic, 1-piece, posterior chamber IOL designed for placement in the capsular bag. Both are made of the same hydrophobic SENSAR material, with a refractive index of 1.47 and an Abbe number of 55. The IOLs have the same overall geometry/dimensions (13 mm overall length and 6 mm optic diameter) as the original parent, the SENSAR 1-piece IOL (model AAB00). The ZXR00 IOL has the same modified prolate (aspheric) design on the anterior optic surface as the ZCB00 IOL to reduce spherical aberration. In addition, the ZXR00 IOL includes a 9-ring diffractive profile on the posterior optic surface, designed to extend the range of vision and to compensate for chromatic aberration of the eye.

### Randomization

Patients were randomly assigned 1:1 to receive either the ZXR00 or the ZCB00 IOL; each patient was to receive the same IOL model in both eyes. Randomization was undertaken by study personnel at each study site by opening sealed, sequentially numbered envelopes containing randomized IOL group information. Study investigators were not masked, but all participants and study evaluators responsible for conducting vision testing remained masked to the type of IOL implanted in each eye during the 6-month study period.

### Surgical Procedure

Before randomization, the choice of the eye to be operated on first was at the discretion of the investigators based on their standard clinical practice (eg, the eye with the worse cataract, poorer corrected distance vision, more severe optical/visual complaints, or eye dominance). Emmetropia (within ±0.5 D) was targeted for all eyes in the study, with the targeted residual refractive error documented.

The investigators used their standard, small-incision phacoemulsification cataract extraction surgical technique. The IOLs were inserted into the capsular bag using the UNFOLDER Platinum 1 Series Implantation System (DK7796 handpiece with the UNFOLDER Platinum 1 Series cartridge, model 1MTEC30) or the ONE SERIES Ultra Implantation System (DK7786 or DK7791 handpiece with the One Series Ultra cartridge, model 1VPR30).

### Clinical End Points

All patients were intended to have bilateral cataract surgery and were to be examined through 6 months postoperatively according to the visit schedule. Distance visual acuities were tested using 100% Early Treatment Diabetic Retinopathy Study (ETDRS) charts at a fixed test distance of 4.0 m under photopic (85 Candelas [cd]/m^2^) lighting conditions. Intermediate visual acuities were tested using 100% ETDRS intermediate charts at a fixed test distance of 66 cm, with and without distance correction, under photopic (85 cd/m^2^) lighting conditions. Near visual acuities were tested using 100% ETDRS near charts at a fixed test distance of 40 cm, with and without distance correction, under photopic (85 cd/m^2^) lighting conditions.

Defocus curve testing was performed on a subset of participants from each IOL group at 8 sites at the 6-month postoperative study examination. Binocular CDVA defocus curves were performed using the electronic Freiburg Visual Acuity and Contrast Test (FrACT).

Monocular corrected distance contrast sensitivity testing was performed using the Vector Vision ETDRS light box and contrast sensitivity charts under 3 lighting conditions: mesopic with glare, mesopic without glare, and photopic with glare. Spectacle wear and other subjective spectacle independence items were assessed by directed, self-reported responses to a binocular subjective questionnaire: the Patient-Reported Spectacle Independence Questionnaire (PRSIQ). Optical/visual symptoms were collected through nondirected, spontaneously reported responses to the open-ended question “Are you having any difficulties with your eyes or vision?”

Safety was assessed by measuring the rate of medical complications or adverse events (AEs). An AE was considered serious if it was an untoward occurrence that may or may not have been related to use of the IOL and that was sight-threatening or life-threatening, resulted in death, required inpatient hospitalization or prolongation of hospitalization, resulted in persistent or significant disability or incapability, or necessitated medical or surgical intervention to prevent permanent impairment to a body structure or function. A Data Safety Monitoring Board reviewed and assessed all reports of serious AEs and, if necessary, discussed these with the reporting investigator without being specific about the IOL type.

### Statistical Analysis

Effectiveness and safety end points were compared for the ZXR00 and ZCB00 IOL cohorts. Analyses were based on the safety population, defined as all first eyes or all patients implanted with either the ZXR00 or the ZCB00 IOL and with data available at the time of analysis. One-sided, 2-sample *t* tests with an alpha level of 0.025 were used for the primary end points of monocular uncorrected intermediate visual acuity (UIVA) and distance-corrected intermediate visual acuity (DCIVA) and the secondary end point of monocular distance-corrected near visual acuity (DCNVA). Clinical significance for these end points was evaluated by examining whether the difference between the ZXR00 and ZCB00 IOL control groups was greater than 25% for the proportion achieving 20/25 or better for intermediate and 20/40 or better for near. Fisher exact test with a 1-sided alpha of 0.025 was used for binocular overall spectacle wear. Nonparametric methods using the lower limit of a 90% confidence limit with a noninferiority margin of −0.15 log units were used for the contrast sensitivity end point. Hierarchical methods (Hodges-Lehman) were used to adjust for multiple statistical comparisons for primary and secondary end points.

Medical complications and AEs were compared with ISO safety and performance end point (SPE) rates for the ZXR00 IOL group using a 1-sided Fisher exact test based on the binomial distribution. Monocular, first-eye, mean CDVA was compared with the ZCB00 IOL group using a noninferiority margin of 0.1 logMAR (1 line). Comparisons between the IOL groups for binocular uncorrected distance visual acuity (UDVA) were performed using 2-sided, 2-sample *t* tests with an alpha set to 0.05.

The sample size was justified based on the primary study end points of monocular UIVA and DCIVA and the requirements for contrast sensitivity testing. With at least 135 evaluable patients in each IOL group, this study had >90% power to detect a difference of ≥0.7 lines in the mean visual acuity between the ZXR00 and ZCB00 IOL groups for UIVA and DCIVA.

## RESULTS

### Patient Disposition

A total of 299 patients were enrolled and implanted with a study IOL across the 15 U.S. clinical study sites. Of the 299 patients enrolled, 148 patients (49.5%) were implanted with the ZXR00 IOL (148 bilaterally implanted) and 151 patients (50.5%) with the ZCB00 IOL (150 bilaterally implanted). All patients were bilaterally implanted except for 1 ZCB00 IOL–implanted patient who was implanted unilaterally due to illness and subsequent death. Of the implanted participants, almost all with ZXR00 (147/148 [99.3%]) and ZCB00 (148/151 [98.0%]) IOLs completed the 6-month follow-up visit.

Patient demographics were similar between the ZXR00 and ZCB00 IOL control groups (Table 1). The mean age of the study participants in both groups was 68 years and more than half of both IOL groups were women. Most patients were White (ZXR00 IOL: 96.6% [143/148]; ZCB00 IOL: 86.1% [130/151]). The remaining patients were Black (ZXR00 IOL: 2.7% [4/148]; ZCB00 IOL: 10.6% [16/151]), Asian (ZXR00 IOL: 0.7% [1/148]; ZCB00 IOL: 2.0% [3/151]), or American Indian/Alaska Native (ZCB00 IOL: 1.3% [2/151]).

**Table 1.** Sex, Mean Age, and Mean Postop SE Refractive Error (Safety Population).

IOL group	Treated (n)	M/F, n (%)	Age (y), mean ± SD	6-mo postop SE, mean ± SD
ZXR00	148	57 (38.5)/91 (61.5)	68.0 ± 7.5	−0.42 ± 0.41
ZCB00	151	65 (43.0)/86 (57.0)	67.9 ± 7.9	−0.36 ± 0.41

ZXR00 = TECNIS Symfony EDoF; ZCB00 = TECNIS monofocal; SE = spherical equivalent

### Monocular Uncorrected and Corrected Visual Acuities

**Figure 1.**
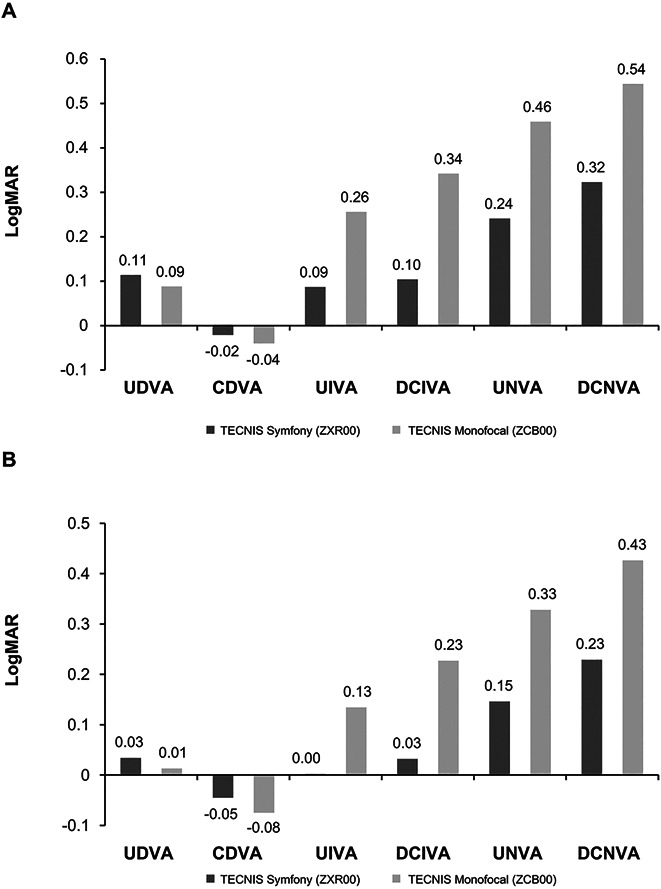
Mean monocular (*A*) and binocular (*B*) visual acuities at the 6-month follow-up.

At the 6-month follow-up, the ZXR00 and ZCB00 IOL groups demonstrated similar mean monocular UDVA (Snellen equivalent 20/25 vs 20/25; difference −0.3 lines; 90% CI, −0.054 to 0.001) and CDVA (20/20 vs 20/20; difference −0.2 lines; 90% CI, −0.036 to −0.003) (Figure 1, A). The difference in monocular mean CDVA between the ZXR00 and ZCB00 IOL groups was −0.2 lines (1 letter), which was within the noninferiority margin of 1 line. A postoperative UDVA of 20/20 or better was observed in 57 (39%) of the 147) ZXR00 IOL–implanted eyes and 70 (47%) of the 148) ZCB00 IOL–implanted eyes, whereas a UDVA of 20/25 or better was observed in 96 (65%) of the 147 ZXR00 IOL–implanted eyes and 106 (72%) of the 148 IOL–implanted ZCB00 eyes. Most of the eyes implanted with ZXR00 (84% [123/147]) and ZCB00 (89% [131/148]) IOLs achieved postoperative CDVA of 20/20 or better, and almost all eyes implanted with ZXR00 (98% [144/147]) and ZCB00 (97% [143/148]) IOLs achieved postoperative CDVA of 20/25 or better.

Figure 1, A also shows mean monocular UIVA, uncorrected near visual acuity (UNVA), DCIVA, and DCNVA for the patients implanted with ZXR00 and ZCB00 IOLs, presented as logMAR values. At the 6-month follow-up, the ZXR00 IOL was associated with significantly better monocular UIVA (Snellen equivalent 20/25 vs 20/36; difference 1.7 lines; *P* < .0001), UNVA (20/35 vs 20/58; difference 2.2 lines; *P* < .0001), DCIVA (20/25 vs 20/44; difference 2.4 lines; *P* < .0001), and DCNVA (20/42 vs 20/69; difference 2.2 lines; *P* < .0001) compared with the ZCB00 IOL. Differences between the ZXR00 and ZCB00 IOL groups were statistically and clinically significant, with a larger proportion (>40%) of the patients implanted with ZXR00 IOL vs ZCB00 IOL achieving predefined targets of 20/25 or better for UIVA and DCIVA and 20/40 or better for DCNVA (Table 2).

**Table 2.** Percentage of Subjects Achieving Targeted Level of Postoperative Monocular Visual Acuity.

End point	ZXR00 IOL (n = 147)	ZCB00 IOL (n = 148)	Difference, %
UIVA 20/25 or better	76.9%	33.8%	43.1
DCIVA 20/25 or better	70.1%	13.5%	56.6
DCNVA 20/40 or better	61.9%	16.2%	45.7

ZXR00 = TECNIS Symfony EDoF; ZCB00 = TECNIS monofocal

### Binocular Uncorrected and Corrected Visual Acuities

**Figure 2.**
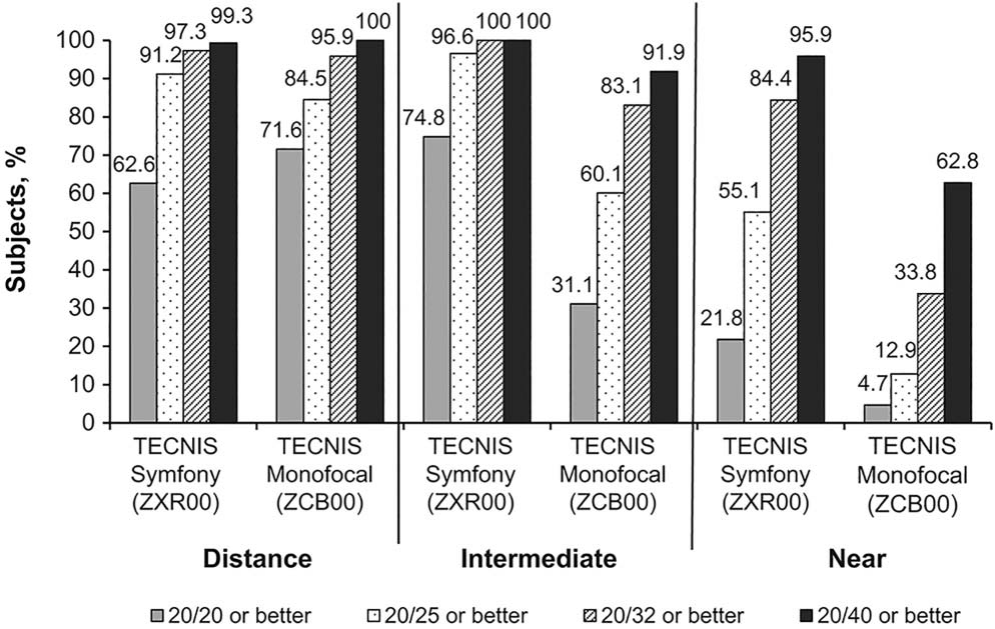
Cumulative binocular uncorrected visual acuity at distance, intermediate, and near distances.

At the 6-month follow-up, the ZXR00 and ZCB00 IOL groups had similar mean binocular UDVA (Snellen equivalent 20/21 vs 20/20; difference −0.2 lines; 90% CI, −0.043 to 0.000; *P* = .1011) and CDVA (20/20 vs 20/16; difference −0.3 lines; 90% CI, −0.046 to −0.015), which was within the noninferiority margin of 1 line (Figure 1, B). More ZXR00 IOL–implanted patients achieved a binocular UDVA of 20/25 or better (91.2% [134/147]) or 20/32 or better (97.3% [143/147]) compared with the ZCB00 IOL–implanted patients (84.5% [125/148] and 95.9% [142/148], respectively) (Figure 2). The 6-month postoperative mean binocular UNVA and UIVA were significantly better for ZXR00 IOL compared with ZCB00 IOL (*P* < .0001) (Table 3). More ZXR00 IOL-implanted patients achieved binocular UNVA of 20/25 or better compared with the ZCB00 IOL–implanted patients (55.1% [81/147] vs 12.8% [19/148]; *P* < .0001) (Figure 2). The results were more dramatic for intermediate visual acuity, with almost all ZXR00 IOL–implanted patients having UIVA of 20/25 or better compared with the ZCB00 IOL–implanted patients (96.6% [142/147] vs 60.1% [89/148]; *P* < .0001) (Figure 2).

**Table 3.** Binocular Uncorrected Visual Acuity at Distance, Intermediate, and Near.

Testing distance	ZXR00 IOL (n = 147)		ZCB00 IOL (n = 148)		logMAR difference	*P* Value
	Mean ± SD	Snellen equivalent	Mean ± SD	Snellen equivalent		
UDVA (4 m)	0.03 ± 0.11	20/21	0.01 ± 0.12	20/20	−0.02	NS (0.1011)
UIVA (66 cm)	0.00 ± 0.09	20/20	0.13 ± 0.14	20/27	0.13	<0.0001
UNVA (40 cm)	0.15 ± 0.11	20/28	0.33 ± 0.17	20/43	0.18	<0.0001

ZXR00 = TECNIS Symfony EDoF; ZCB00 = TECNIS monofocal; NS = not significant

### Spherical Equivalent Refraction and Refractive Cylinder

No statistically significant difference was found between IOL groups for mean target spherical equivalent (SE), mean SE at 6 months, and mean refractive cylinder at 6 months (all *P* > .05). Postoperative mean SE was slightly myopic for both IOL groups, with a mean of −0.42 (±0.41) for the ZXR00 IOL group and −0.36 (±0.41) for the ZCB00 IOL group; the mean target SEs were −0.20 (±0.15) and −0.19 (±0.15), respectively. The mean refractive cylinder was 0.39 (±0.38) for the ZXR00 IOL group and 0.42 (±0.39) for the ZCB00 IOL group.

**Figure 3.**
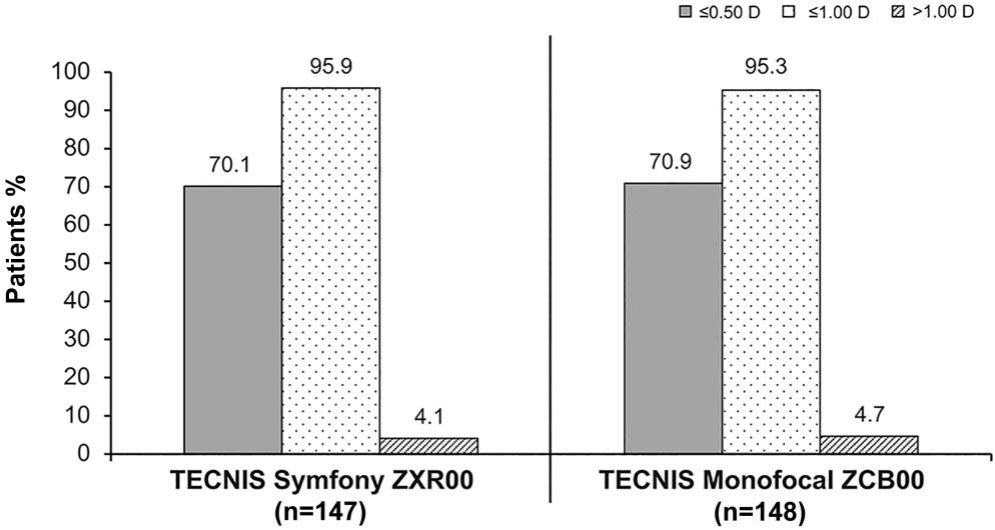
Postoperative absolute manifest refraction spherical equivalent relative to the intended emmetropic target at the 6-month follow-up.

**Figure 4.**
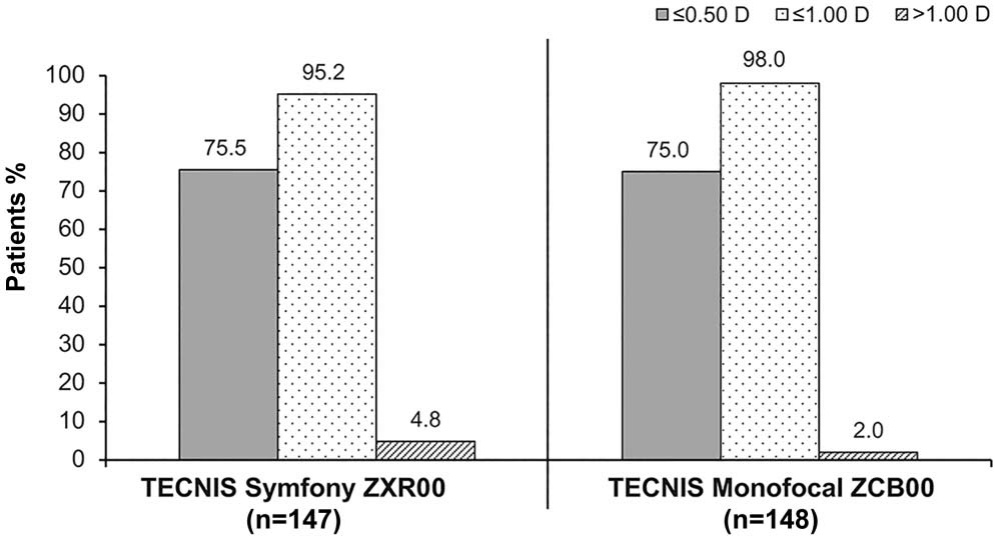
Absolute refractive cylinder at the 6-month follow-up.

The 6-month postoperative absolute manifest SE relative to the intended emmetropic target was within ±0.50 D of emmetropia in 103 (70.1%) of the 147 ZXR00 IOL–implanted first eyes and 105 (70.9%) of the 148 ZCB00 IOL–implanted first eyes (70.9%); absolute manifest SE was within ±1.00 D of emmetropia in 141 (95.9%) of the 147 ZXR00 IOL–implanted first eyes and in 141 (95.3%) of the 148 ZCB00 IOL–implanted first eyes (Figure 3). The 6-month postoperative refractive cylinder was within ±0.50 D of emmetropia in 111 (75.5%) of the 147 ZXR00 IOL–implanted first eyes and in 111 (75.0%) of the 148 ZCB00 IOL–implanted first eyes; refractive cylinder was within ±1.00 D of emmetropia in 140 (95.2%) of the 147 ZXR00 IOL–implanted first eyes and in 145 (98.0%) of the 148 of ZCB00 IOL–implanted first eyes (Figure 4). No statistically significant differences were observed between IOL groups for the proportions of eyes within ±0.5 D and ±1.0 D for SE (*P* = .8989 and *P* = 1.0000, respectively) or for refractive cylinder (*P* = 1.0000 and *P* = .2177, respectively).

### Defocus Curves

**Figure 5.**
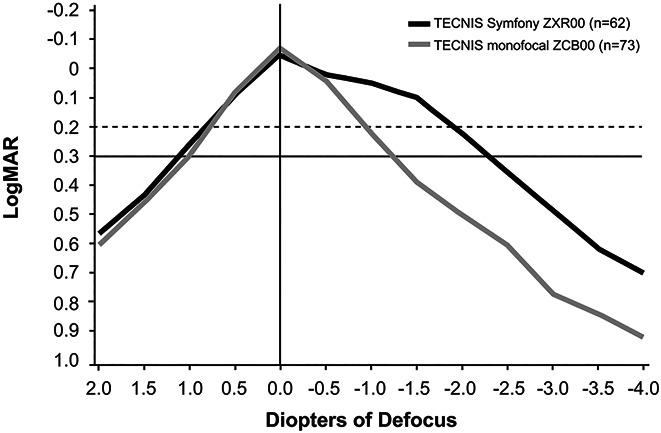
Binocular CDVA defocus curves and visual acuity means.

Binocular defocus curves revealed an approximately 1.0 D greater range of defocus by ZXR00 IOL vs ZCB00 IOL (Figure 5). The mean binocular acuities were 20/32 or better for the ZXR00 IOL group through −2.0 D (50 cm). The ZXR00 IOL group binocular defocus curves showed a 1-line to 2-line acuity improvement over the ZCB00 IOL group through 4.0 D of defocus. When visual acuity means from the standard ETDRS acuity testing are plotted on the defocus chart at far, intermediate, and near distances, a similar difference of 1 to 2 lines of acuity is seen in favor of the ZXR00 IOL group from −1.0 to −4.0 D of focus over the ZCB00 IOL group.

### Patient-Reported Outcomes

#### Spectacle Wear

**Figure 6.**
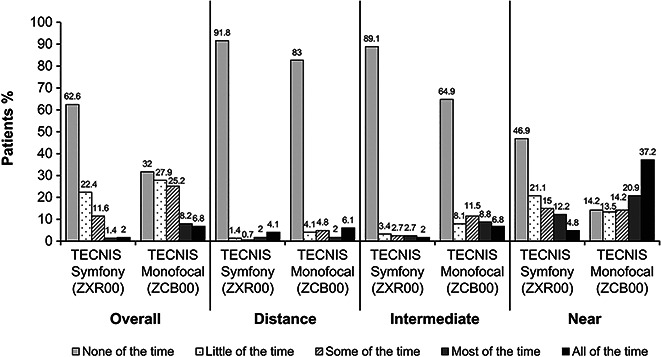
Frequency of spectacles/contacts wear for overall, distance, intermediate, and near vision during the last 7 days as reported 6 months postoperatively.

Spectacle wear was significantly lower for patients receiving the ZXR00 IOL compared with those receiving the ZCB00 IOL. At the 6-month postoperative visit, 125 (85%) of 147 patients with bilateral ZXR00 IOL vs 88 (59.9%) of 147 patients with ZCB00 IOL reported wearing spectacles or contact lenses none of the time or a little of the time for overall vision within the last 7 days (*P* < .0001). A significantly higher proportion of ZXR00 IOL–implanted patients reported wearing spectacles or contacts none of the time at the 6-month postoperative visit, compared with the ZCB00 IOL group (62.6% [92/147] vs 32.0% [47/147]; *P* < .0001) (Figure 6).

#### Visual Symptoms

Spontaneous nondirected reports of image quality were excellent in both ZXR00 and ZCB00 IOL groups, with only 6 (4.1%) of 147 patients and 8 (5.4%) of 148 patients reporting blurred vision overall, respectively. Visual symptoms typically associated with presbyopia-correcting IOLs were low for patients implanted with the ZXR00 IOL and were only slightly higher than reports from patients implanted with the ZCB00 IOL (Table 4). At 6 months, the most common spontaneously reported optical/visual symptoms were halos and starbursts for the ZXR00 IOL. Night glare difficulty was uncommon with both the ZXR00 and ZCB00 IOL groups (2.7% [4/147] and 0% [0/148], respectively).

**Table 4.** Optical/Visual Symptoms at 6 Months Postop (Nondirected).

Symptom	ZXR00 IOL (n = 147)				ZCB00 IOL (n = 148)			
	None, % (n)	Mild, % (n)	Mod, % (n)	Severe, % (n)	None, % (n)	Mild, % (n)	Mod, % (n)	Severe, % (n)
Night glare	97.3 (143)	0.7 (1)	2.0 (3)	0.0 (0)	100 (148)	0.0 (0)	0.0 (0)	0.0 (0)
Halos	83.7 (123)	6.1 (9)	7.5 (11)	2.7 (4)	98.6 (146)	0.7 (1)	0.0 (0)	0.7 (1)
Starburst	91.2 (134)	4.1 (6)	3.4 (5)	1.4 (2)	98.6 (146)	0.7 (1)	0.7 (1)	0.0 (0)

ZXR00 = TECNIS Symfony EDoF; ZCB00 = TECNIS monofocal

#### Quality of Nighttime Vision

At the 6-month follow-up, most patients implanted with the ZXR00 or ZCB00 IOL reported having good vision quality for far (78.8% [115/146] and 83.6% [122/146], respectively), intermediate (76.0% [111/146] and 78.1% [114/146], respectively), and near (67.8% [99/146] and 75.3% 110/146], respectively) distances under nighttime outdoor lighting conditions.

### Contrast Sensitivity

**Figure 7.**
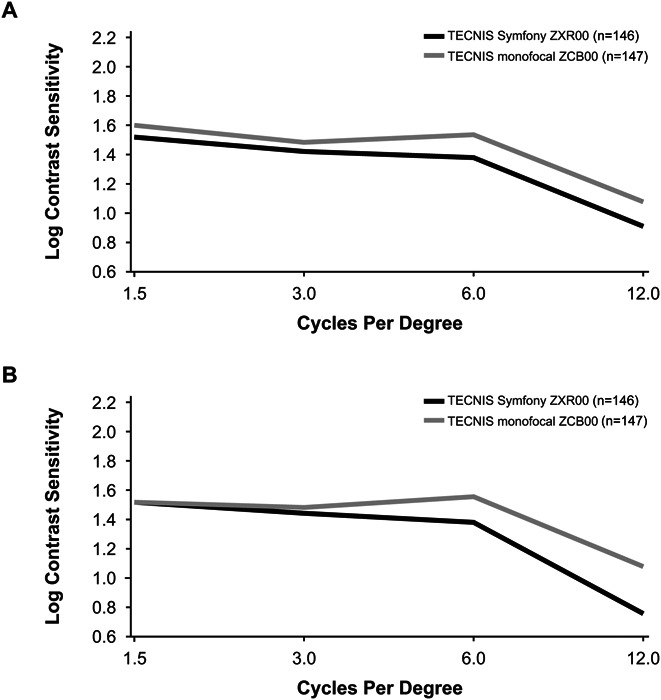
Median monocular contrast sensitivity at 6 months under mesopic conditions with (*A*) and without (*B*) glare.

The median values for monocular best-corrected contrast sensitivity for ZXR00 and ZCB00 IOLs were not statistically different at 1.5 and 3.0 cycles per degree under either mesopic or mesopic with glare lighting conditions (Table 5; Figure 7, A and B). At 6.0 and 12.0 cycles per degree, the median difference between IOL groups exceeded −0.15 log units for mesopic with glare and the lower limit of the 90% CI was below the noninferiority margin of −0.15 log units for with and without glare; however, the median difference and lower limit of the 90% CI were within −0.30 log units (the difference typically considered clinically significant loss when occurring at 2 or more spatial frequencies).

**Table 5.** Median Monocular Best-Corrected Contrast Sensitivity at 6 Months Under Mesopic Lighting Conditions.

Spatial frequency	IOL	n	Mesopic without glare, logMAR			Mesopic with glare, logMAR		
			Median	Lower 90% CI	Upper 90% CI	Median	Lower 90% CI	Upper 90% CI
1.5 cpd	Symfony	146	1.520	1.445	1.595	1.520	1.445	1.520
	Monofocal	147	1.595	1.520	1.595	1.520	1.445	1.595
	Difference		−0.075	−0.075	0.000	0.000	−0.075	1.000
3.0 cpd	Symfony	146	1.415	1.340	1.475	1.445	1.340	1.560
	Monofocal	147	1.490	1.475	1.490	1.490	1.485	1.560
	Difference		−0.075	−0.145	0.000	−0.070	−0.085	0.000
6.0 cpd	Symfony	146	1.380	1.380	1.465	1.380	1.380	1.465
	Monofocal	147	1.540	1.465	1.550	1.550	1.540	1.625
	Difference		−0.145	−0.170	−0.075	−0.160	−0.235	−0.085
12.0 cpd	Symfony	146	0.910	0.845	0.995	0.760	0.610	0.910
	Monofocal	147	1.080	0.995	1.080	10.80	0.995	1.080
	Difference		−0.085	−0.170	0.000	−0.155	−0.290	0.000

### Safety

The most frequently reported medical complications/AEs 1 day postoperatively for both IOL groups for first eyes were cells (ZXR00 IOL: 79.7% [118/148]; ZCB00 IOL: 78.8% [119/151]), flare (ZXR00 IOL: 16.9% [25/148]; ZCB00 IOL: 19.2% [29/151]), and corneal edema (ZXR00 IOL: 27% [40/148]; ZCB00 IOL: 26.5% [40/151]), which diminished over time to minimal levels by the 1-month visit in both IOL groups. Similar results were found for second eyes for both groups. Nd:YAG capsulotomy rates were low in both groups at 6 months, with 14 (4.7%) of 296 ZXR00 IOL and 5 (1.7%) of 301 ZCB00 IOL control eyes requiring the procedure during the study.

Overall, 4 (2.7%) of the 148 ZXR00 IOL–implanted patients experienced serious AEs during the study and none (0%) experienced device-related or unanticipated events. Serious ZXR00 IOL AEs were as follows: cystoid macular edema (2 eyes), hypopyon/endophthalmitis (1 eye), pupillary capture (1 eye), and secondary surgical intervention (antibiotic injections [2 eyes]). Serious AEs were more common in the ZCB00 IOL group (6.0%, 9/151), comprising cystoid macular edema (5 eyes), anterior ischemic optic neuropathy (1 eye), and secondary surgical intervention (6 eyes: fragment removals [2 eyes], treatment injections for medical complications [2 eyes], epiretinal membrane peel [1 eye], and stromal puncture for anterior basement membrane dystrophy [1 eye]). The incidence rates for the ZXR00 IOL compared favorably with the specified ISO SPE rates as the observed rates for ZXR00 were within the specified ISO SPE rates or not statistically significantly higher.

## DISCUSSION

This clinical investigation evaluated the effectiveness and safety of an extended depth of focus lens, the TECNIS Symfony EDoF IOL, model ZXR00, compared with the monofocal control IOL, the aspheric TECNIS 1-piece IOL, model ZCB00. The IOL delivers well-focused vision over an enhanced range, thus providing good distance vision and improved intermediate and near vision compared with monofocal IOLs.^10–12^ In addition, the ZXR00 IOL maintained high-contrast visual acuity and patient satisfaction after cataract surgery or refractive lens exchange. The clinical study results achieved at 6 months postoperatively demonstrated improved uncorrected and distance-corrected intermediate and near vision, an increased depth of focus, and decreased spectacle wear in participants who received the ZXR00 IOL compared with the monofocal control IOL. End points for distance visual acuity showed comparable performance for the ZXR00 IOL and the monofocal control IOL. Safety measures showed good contrast sensitivity, typical optical/visual symptoms, and low rates of AEs.

Theoretical attributes and clinical outcomes of the ZXR00 IOL have been previously published.^10–14^ An analysis comparing the ZXR00 and ZCB00 monofocal IOLs in 80 eyes reported significantly better uncorrected monocular and binocular distance, intermediate, and near visual acuities (*P* ≤ .013) for the ZXR00 IOL group vs the monofocal IOL group, and no statistically significant between-group differences were observed in contrast sensitivity or optical quality parameters (*P* > .05).^10^ Similarly, in a multicenter study of 411 patients who received bilateral implantations with the ZXR00 IOL and assigned to a micromonovision arm (n = 112; residual myopia was targeted in the nondominant eye between 0.50 D and 0.75 D) or a group targeted for emmetropia, mean binocular UDVA (0.03 ± 0.10 logMAR), UIVA (0.12 ± 0.16 logMAR), and UNVA (0.19 ± 0.17 logMAR) were similar to those reported in this study, with the UNVA approximately 1 line better for the micromonovision group vs the group targeted for emmetropia.^12^ In a prospective noncomparative case series of 52 eyes, bilateral implantation of the TECNIS Symfony IOL was also associated with excellent UDVA and UIVA (<0.1 logMAR) and acceptable UNVA (<0.3 logMAR).^13^ Furthermore, comparison of 2 EDoF IOLs, TECNIS Symfony and IC-8, in the 6-month prospective randomized trial of 36 patients, also reported excellent UDVA, good UNVA and UIVA, and high patient satisfaction regarding visual acuity without spectacles/contact lenses for both EDoF IOLs.^14^ Studies evaluating multifocal IOLs (TECNIS ZM900 and TECNIS ZKB00 [Johnson & Johnson Vision]) also reported positive distance, intermediate, and near visual acuities, which were comparable with visual acuity outcomes observed with the ZXR00 IOL in this study.^15^ Furthermore, visual symptoms of halo and glare reported with the ZXR00 IOL were substantially lower than those reported for the ZM900 and ZKB00 multifocal IOLs, meeting the intended design concept of reduced visual symptoms with the ZXR00 IOL.^15,16^

The PRSIQ is a new tool primarily developed for assessing spectacle independence in patients after cataract surgery.^17^ It is the only validated questionnaire for posterior chamber IOLs, and it aims to determine the need, wear, and frequency of spectacle or contact lens use during the 7 days immediately prior to the survey. The PRSIQ was effectively used in this study to determine spectacle use postoperatively and showed that a significantly higher proportion of patients implanted with ZXR00 IOL reported not wearing spectacles or contact lens 6 months postoperatively (*P* < .0001).

Positive visual outcomes with trifocal IOLs, which combine 2 diffractive profiles to improve vision across all spectrums, have been previously reported for the AT LISA tri 839MP (Carl Zeiss Meditec AG), FineVision Micro F (Physiol S.A.), and AcrySof IQ PanOptix (Alcon Laboratories, Inc.) IOLs.^18–23^ These smaller, uncontrolled studies reported binocular uncorrected visual acuities 3 to 6 months postoperatively that ranged from −0.06 to 0.02 logMAR for distance (4 m), 0.00 to 0.32 logMAR for intermediate (70 to 80 cm), and 0.02 to 0.15 logMAR for near (40 cm).^18–21^ Defocus curves for the FineVision Micro F trifocal IOL showed 2 peaks that corresponded with distance and near acuities, with a smaller drop between these peaks when compared with defocus curves for a bifocal IOL.^19^ Photic phenomena reports were evaluated by different methods in each of the studies, with the highest complaints being glare and halo, at similar or lower levels than are seen in other multifocal IOLs.^19,21^ Compared with the findings of these trifocal studies, the ZXR00 IOL showed similar binocular uncorrected visual acuities and the unique extended range of vision feature, shown in the monotonically decreasing defocus curve. Indeed, a small 6-month, prospective, randomized study comparing 2 trifocal IOLs (AcrySof IQ PanOptix and FineVision Micro F) and the TECNIS Symfony IOL also reported similar binocular UDVA and UIVA outcomes, low incidence rates of photic phenomena (<1%), and a high level of spectacle independence (90% overall).^23^ Furthermore, a 6-month study of 411 patients bilaterally implanted with the TECNIS Symfony IOL demonstrated that loss of binocular UDVA, UIVA, or UNVA did not exceed 1 line and was not clinically relevant in eyes with residual cylinders up to 0.75 D.^24^ These findings highlight that the TECNIS Symfony IOL may provide better tolerance to postoperative refractive errors (ie, residual astigmatism), which is an important factor for ensuring patient satisfaction.^24^

Binocular UDVA was similar between the ZXR00 and ZCB00 IOL control groups, demonstrating the ability of ZXR00 IOL to provide good distance visual acuity. Pedrotti et al. found similar positive distance visual acuity results with the Symfony IOL.^12^ In their study, the authors reported significantly improved mean monocular UDVA in the Symfony IOL group (0.08 ± 0.12 logMAR) compared with monofocal IOLs (0.14 ± 0.14 logMAR, *P* = .013) at the 3-month follow-up.^12^ Binocular UDVA of 0.20 logMAR or better (Snellen 20/30) was observed with both Symfony (0.00 ± 0.09 logMAR) and monofocal (0.03 ± 0.11 logMAR) IOLs.^12^ In another study comparing the Symfony IOL with trifocal IOLs, the Symfony IOL was associated with significantly better mean UDVA compared with the AT LISA tri 839 and PhysIOL FineVision IOLs (1.01 [−0.004 logMAR] vs .96 [0.018 logMAR] and 0.95 [0.022 logMAR]; *P* = .048 and *P* = .006, respectively).^8^ These findings indicate that the Symfony IOL provides good-quality distance vision.

Some noteworthy findings of the study were those observed for the contrast sensitivity testing and defocus curves. A slight but not clinically significant reduction in monocular contrast sensitivity at higher frequencies was found for the ZXR00 IOL compared with the aspheric control IOL, an IOL known to have contrast superior to nonaspheric monofocal IOLs.^25,26^ The optical design of the ZXR00 IOL includes a diffractive profile on the posterior optic surface designed to reduce the chromatic aberrations of the eye. Preclinical data predict that the correction of spherical and chromatic aberration is expected to counteract the change in contrast that accompanies an extension of depth of focus such that overall contrast is maintained comparable with that of a low-dispersion monofocal IOL that corrects spherical aberration only.^10^

Although there were differences in contrast sensitivity between the ZXR00 IOL and the aspheric monofocal control IOL, it should be noted that the control IOL in this study is a monofocal IOL that fully corrects spherical aberration and minimizes chromatic aberration, yielding higher contrast than standard spherical IOLs, particularly those with higher levels of chromatic dispersion.^27^ In addition, contrast sensitivity tests in this study were conducted monocularly, and it is possible that differences in contrast outcomes between IOL groups may be reduced when testing binocularly, because binocular summation helps patients achieve contrast values closer to the retinal threshold limits.^2,28,29^

Another finding of interest in this study concerns the methods used to evaluate defocus curves, which illustrate the extended depth of focus of the Symfony optic design. In this study, distance, intermediate, and near visual acuities were measured with the ETDRS chart, and defocus testing was performed using FrACT. The FrACT test uses Landolt C optotype and a thresholding method to measure visual acuity, whereas the ETDRS test is a standard method that has been optimized for efficient clinical visual acuity testing. Both ETDRS and FrACT are valid visual acuity measurement systems that showed generally good correlation for visual acuity testing.^30,31^ However, ETDRS visual acuity results tested at a specific distance may not be directly comparable with FrACT defocus results obtained through minus IOLs because of the difference in measurement methods.^32^ In this clinical study, fatigue due to the order of testing and the long duration of FrACT defocus testing may have further contributed to lower visual acuities in the FrACT defocus test compared with the ETDRS real distance test. Nonetheless, the differences seen between ETDRS and FrACT were similar for both Symfony and monofocal control IOL groups and across all sites. As lower acuities were found with the FrACT defocus method compared with ETDRS direct testing, the use of the FrACT system for the defocus testing may provide a more conservative estimate of defocus diopter range with visual acuity of 20/32 or better compared with using ETDRS for defocus testing. In this study, the defocus curve for ZXR00 IOL showed that near vision decreased below the 20/40 level at 2.5 D (40 cm), after which the near vision continued to decline monotonically. Because this reduction in visual acuity with the FrACT test was observed with both Symfony and monofocal control IOLs, the difference in depth of focus between IOLs is robust.

Patient-reported outcomes indicated that good vision under nighttime outdoor lighting conditions was reported by most patients receiving the ZXR00 IOL. Although halo and starburst effects were more common with ZXR00 IOL than monofocal IOLs, most cases were mild or moderate. These findings highlight that the ZXR00 IOL provides good quality nighttime vision with a low incidence of night vision symptoms.

Chromophores that selectively reduce high-energy, short-wavelength light have the potential to reduce night vision symptoms.^33–36^ IOLs with violet filtering have been developed to further reduce these symptoms. In a randomized study of 240 patients comparing a violet light–filtering monofocal IOL with a colorless IOL, comparable CDVA was observed between the violet light–filtering and colorless IOLs, with similar proportions of patients achieving 20/20 or better and 20/40 or better in both first (82.4% and 100% vs 86.6% and 100%, respectively) and second (84.9% and 99.2% vs 92.5% and 100%, respectively) eyes.^35^ Compared with the colorless IOL group, significantly greater proportions of patients in the violet light–filtering IOL group reported no difficulty in driving during daytime (98.1% vs 91.7%; *P* = .033) or at nighttime (52.8% vs 44.4%; *P* = .017) and no frustration with vision (89.8% vs 79.8%; *P* = .0325). Taken together, these findings indicate that the use of violet light–filtering IOLs can improve visual functioning while maintaining visual acuity and contrast sensitivity. In particular, improvements in visual function related to daily activities (eg, nighttime driving) support the use of violet light–filtering technology in presbyopia-correcting IOLs to provide patients receiving these IOLs with potentially greater benefits regarding activities associated with scotopic vision.

Findings of this randomized, controlled, masked clinical study provide practitioners and patients with information about the safety and effectiveness of this unique IOL technology. The ability of EDoF IOLs, such as the ZXR00 IOL, to provide a more natural range of vision provides the opportunity for a personalized approach to IOL selection in which EDoF and multifocal IOLs can be used in tandem to achieve a range of vision and nighttime vision symptom profile suitable for each individual patient. Recently, blended implantation of the ZXR00 IOL in the dominant eye and +3.25 low-add multifocal IOL (Tecnis ZLB00) in the nondominant eye provided excellent uncorrected visual acuity at near, intermediate, and far distances with minimal ocular symptoms.37 Blended implantation of the ZXR00 IOL and a diffractive multifocal IOL (TECNIS ZMB00) also exhibited better performance regarding quality of vision for long, intermediate, and short distances, compared with a trifocal IOL (Acrysof IQ Panoptix TFNT00).^38^

The results for defocus testing in this study were conservative because of the aforementioned testing differences but provide a basis for potential functional performance that can be expected with this IOL design. Understanding the differences inherent to clinical testing can also help with future study designs and interpretation of results. To expand the knowledge base for this IOL technology, future studies are needed in both real-world and clinical settings.

In conclusion, clinical results from this study at 6 months postoperatively demonstrated that the TECNIS Symfony IOL provided patients with improved uncorrected intermediate and near visual acuity, comparable distance visual acuity, an increased depth of focus, and decreased use of spectacles when compared with the monofocal control IOL. Review of safety outcomes with the new IOL design revealed no significant safety concerns, acceptable contrast sensitivity and optical/visual symptoms, and low rates of AEs.

**WHAT WAS KNOWN**

- Cataract surgery with monofocal IOLs often requires patients to wear spectacles for reading or performing other near tasks, even if a monovision option is selected.
- Patients with multifocal IOLs are able to read and perform other near tasks without spectacles, but they sometimes experience dysphotopsia (eg, halos), particularly at night, and have limited intermediate ability (eg, they may need spectacles to work on a computer).

**WHAT THIS PAPER ADDS**

- The TECNIS Symfony IOL, model ZXR00, was a safe and effective option in patients undergoing cataract surgery, providing improved uncorrected and distance-corrected intermediate and near vision, an increased depth of focus, and decreased spectacle wear when compared with the TECNIS 1-piece IOL.
